# Quantum biological convergence: quantum computing accelerates KRAS inhibitor design

**DOI:** 10.1038/s41392-025-02239-2

**Published:** 2025-05-14

**Authors:** Taeho Kwon, Hakjin Kim

**Affiliations:** 1 Primate Resources Center, Futuristic Animal Resource and Research Center, Korea Research Institute of Bioscience and Biotechnology (KRIBB), Jeongeup-si, Jeonbuk Republic of Korea; 2 Advanced Bioconvergence Department, KRIBB School, Korea National University of Science and Technology (UST), Daejeon, Republic of Korea; 3 In Quantio, Songdo Consulting Group, Incheon, Republic of Korea

**Keywords:** Computational biology and bioinformatics, Lung cancer

In a recent study published in *Nature Biotechnology*, Mohammad Ghazi Vakili et al. applied quantum computing and generative machine learning—specifically Quantum Circuit Born Machines (QCBMs) and Long Short-Term Memory (LSTM) networks—to efficiently explore high-dimensional chemical space and identify structurally novel KRAS inhibitors.^1^ This research highlights how quantum-enhanced AI (artificial intelligence), when supported by substantial pre-existing data, can contribute to the discovery of inhibitors for challenging targets such as KRAS.^1^

KRAS is a frequently mutated oncogene in lung, colorectal, and pancreatic cancers, yet its direct inhibition remains challenging due to its smooth surface and absence of deep binding pockets. While covalent inhibitors such as sotorasib (now an approved therapy) have demonstrated promise targeting KRAS-G12C, and inhibitors such as MRTX1133 are undergoing clinical development for KRAS-G12D, significant hurdles remain in achieving broad-spectrum inhibition across multiple KRAS variants, including G12V.^2^ This highlights the urgent need for complementary strategies capable of expanding the druggable landscape.

The study used a QCBM, a quantum generative model that employs quantum superposition and entanglement to efficiently sample complex molecular distributions. Integrated with an LSTM network, a powerful deep-learning algorithm frequently used for sequential data modeling, this hybrid computational approach enables the de novo design of drug-like molecules featuring optimized pharmacological properties. By combining these computational techniques, researchers successfully navigated the vast chemical space and identified molecules exhibiting high predicted binding affinity, selectivity, and synthetic accessibility. Unlike traditional drug discovery methodologies that rely on high-throughput screening and iterative optimization processes, this quantum-enhanced AI framework allows rationally designed inhibitors specifically tailored for KRAS mutants.

The quantum-inspired generative model screened over one million molecules, identifying 15 potential inhibitors predicted to bind KRAS with high affinity. Among these, ISM061-018-2 and ISM061-022 were selected for experimental validation. SPR (surface plasmon resonance) assays confirmed measurable binding of these inhibitors to KRAS, with affinities in the micromolar range, indicating direct target engagement. Although the SPR results did not reflect high-affinity binding, subsequent cell-based assays demonstrated functional inhibition of KRAS signaling and reduced proliferation of KRAS-mutant cancer cell lines, suggesting potential intracellular activity. The ability of these molecules to target multiple KRAS mutants—including G12D, G12C, and G12V—suggests their potential as pan-KRAS inhibitors, addressing a critical gap in targeted cancer therapies (Fig. 1).

**Fig. 1:**
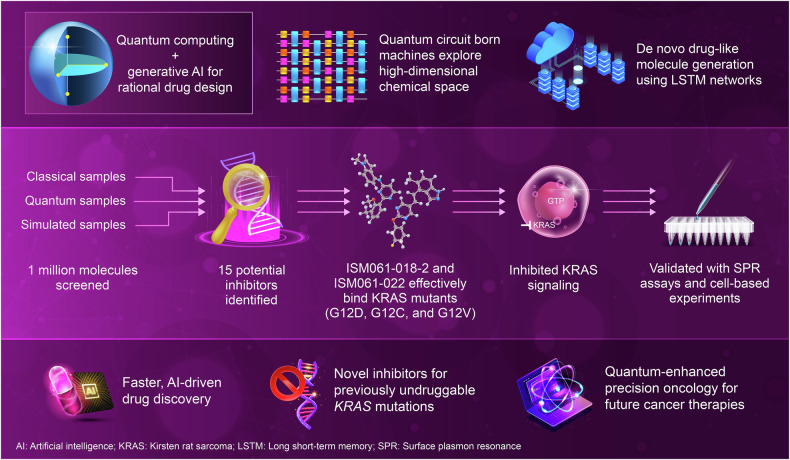
Quantum-enhanced AI (artificial intelligence) identifies candidate KRAS inhibitors. A hybrid AI framework integrating QCBMs (quantum circuit born machines) and LSTM (long short-term memory) networks was employed to generate and screen over 1 million molecules for potential KRAS inhibition. Among the 15 selected candidates, ISM061-018-2 and ISM061-022 were evaluated further. These compounds demonstrated measurable binding to KRAS mutants (G12D, G12C, and G12V) in SPR (surface plasmon resonance) assays, with affinities in the micromolar range. In KRAS-mutant cancer cell lines, both molecules exhibited moderate inhibition of cell proliferation. Although these results indicate preliminary activity and justify further investigation, this study highlights the potential for quantum-enhanced generative models to support early-stage drug discovery in the context of well-characterized oncogenic targets. This figure was created using Editage (www.editage.com)

One primary difficulty in KRAS targeting has been its highly dynamic conformational landscape, rendering identification of stable binding sites particularly challenging. Traditional drug discovery approaches largely rely on classical force-field-based docking simulations, which struggle to capture the full complexity of KRAS conformational flexibility. Although the model may eventually identify non-obvious chemical patterns, the current implementation does not directly incorporate structural data from the target during generation but rather evaluates potential binding of compounds post hoc. Thus, claims regarding the exploration of non-intuitive chemical interactions should be interpreted with caution.^3^ With its quantum computing integration, the generative modeling system facilitates the discovery of structurally novel inhibitors, avoiding redundancies frequently present in traditional compound libraries. As a result, this approach significantly reduces risks associated with intellectual property conflicts while broadening chemical diversity in potential therapeutics.

The findings of this study have significant implications for the future of precision oncology and rational drug design, as the application of quantum-enhanced AI in small-molecule drug discovery offers several key advantages.^4,5^ First, it substantially accelerates drug development timelines by streamlining the traditionally time-consuming process of identifying and optimizing lead compounds through rapid generation and screening of drug-like molecules. Second, it improves success rates through AI-driven predictive modeling, enabling researchers to pre-screen compounds for optimal ADME-Tox properties (absorption, distribution, metabolism, excretion, and toxicity), thereby reducing the likelihood of late-stage failures. Third, quantum computing facilitates the targeting of challenging proteins such as KRAS, previously considered intractable due to their lack of traditional binding sites, by enabling exploration of novel binding modes that could pave the way for first-in-class inhibitors. However, this demonstration relied upon extensive available prior data on KRAS—such as 650 known inhibitors, 250,000 compounds from a structure-based virtual screen, and over 850,000 similar molecules—and such data density is seldom available for truly undrugged targets. Moving forward, broader application of this strategy will necessitate overcoming these data limitations. Further refinement of the quantum generative model could enhance predictive accuracy, and the incorporation of AI-driven molecular docking simulations may improve binding affinity estimations. Additionally, integrating fragment-based drug discovery and SBDD approaches could further enhance the potency and selectivity of KRAS inhibitors.

The combination of quantum computing and AI in drug discovery represents a transformative advancement, providing a scalable and efficient platform for small-molecule design. Although this approach holds promise for addressing challenging drug targets, its effectiveness largely depends on the availability of high-quality molecular data, as exemplified by the extensive KRAS compound datasets used in the original study. Nevertheless, the current findings also highlight how hybrid quantum-classical models can accelerate early-stage discovery and how advancing computational capabilities alongside improved implementation of structure-based methods can potentially reshape precision oncology and expand the landscape of actionable targets in cancer therapy.

## References

[1] Ghazi Vakili, M. et al. Quantum-computing-enhanced algorithm unveils potential KRAS inhibitors. *Nat. Biotechnol*. 10.1038/s41587-024-02526-3 (2025). 10.1038/s41587-024-02526-3 PMC12700792 39843581

[2] Wang, X. et al. Identification of MRTX1133, a noncovalent, potent, and selective KRAS(G12D) inhibitor. *J. Med. Chem.* **65**, 3123–3133 (2022). 34889605 10.1021/acs.jmedchem.1c01688

[3] Yan, X. et al. Multiscale calculations reveal new insights into the reaction mechanism between KRAS(G12C) and alpha, beta-unsaturated carbonyl of covalent inhibitors. *Comput. Struct. Biotechnol. J.* **23**, 1408–1417 (2024). 38616962 10.1016/j.csbj.2024.03.027 PMC11015740

[4] Batra, K. et al. Quantum machine learning algorithms for drug discovery applications. *J. Chem. Inf. Model.* **61**, 2641–2647 (2021). 34032436 10.1021/acs.jcim.1c00166 PMC8254374

[5] Santagati, R. et al. Drug design on quantum computers. *Nat. Phys.* **20**, 549–557 (2024).

